# Open-Air Growth of Polymer Brushes by Surface-Initiated PhotoATRP under Red-Light Irradiation

**DOI:** 10.1021/acsami.5c08584

**Published:** 2025-06-18

**Authors:** Yuwen Zhang, Alessio Lo Bocchiaro, Xiaolei Hu, Carlos Pavon, Cristian Pezzato, Krzysztof Matyjaszewski, Francesca Lorandi, Edmondo M. Benetti

**Affiliations:** † Laboratory for Macromolecular and Organic Chemistry, Department of Chemical Sciences, University of Padova, Via Marzolo 1, Padova 35131, Italy; ‡ Department of Chemistry, 6612 Carnegie Mellon University, 4400 Fifth Avenue, Pittsburgh, Pennsylvania 15213, United States

**Keywords:** polymer brushes, SI-ATRP, open air polymerizations, methylene blue, surface functionalization

## Abstract

The translation of polymer brushes into technologically relevant coatings hinges on the development of scalable and robust fabrication strategies that are tolerant of environmental conditions. Surface-initiated photoinduced atom transfer radical polymerization (SI-photoATRP) has emerged as a powerful tool for synthesizing functional brushes with precise control over their architectural parameters. However, traditional SI-photoATRP requires high-energy light and confined setups to mitigate oxygen inhibition within nondeoxygenated mixtures, limiting substrate versatility and process scalability. Herein, we report a red-light-driven SI-photoATRP process enabled by a catalytic system composed of methylene blue (MB^+^) and a Cu-based ATRP catalyst, which achieves efficient polymer brush growth under fully open-air conditions. Systematic variation of reaction parameters—including light intensity, composition of the catalytic system, and solvent—enabled rapid growth of compositionally different brushes with high and tunable thickness. The deep penetration capability of red light was exploited to decorate microporous three-dimensional materials with polymer brushes. Spatially defined brush growth was demonstrated by shifting the wavelength of light irradiation, alternatively stimulating surface-initiated polymerization in the outer volumes of the support or uniformly across the entire microporous material.

## Graphical abstract

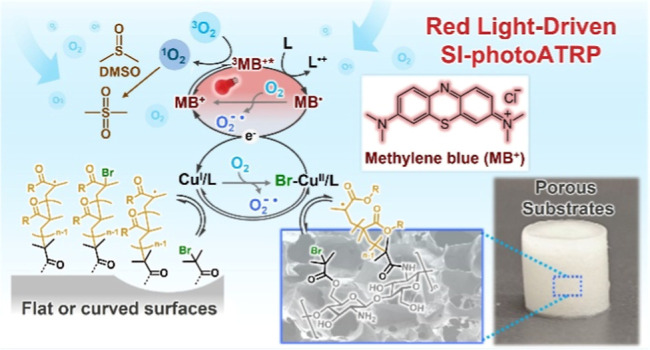

## 1 Introduction

Translation of polymer brushes into technologically relevant coating materials requires robust and scalable fabrication processes, which necessarily encompass the development of surface-initiated controlled polymerization techniques that can be easily performed within industrially relevant settings.

Surface-initiated reversible deactivation radical polymerizations (SI-RDRPs) represent the major class of techniques for synthesizing polymer brushes, giving access to a broad variety of functional brush coatings with fine control over polymer structural parameters, including thickness (*i.e.*, molar mass), dispersity (*Đ*), grafting density (σ), and polymer architecture/topology.^1^

Among the main requirements for developing SI-RDRP processes that are compatible with the large-scale synthesis of brushes, a high tolerance toward environmental conditions is probably the most stringent.^2–5^ Processes that are compatible with nondegassed reaction mixtures and/or exposure to the environment permit a steady brush growth while avoiding radical recombination with molecular oxygen. To this aim, different SI-RDRP techniques have been developed, typically exploiting oxygen scavengers present in the polymerization mixture, including zerovalent metals,^6–9^ sacrificial catalysts/cocatalysts,^10–13^ and enzymes.^14,15^

Among the SI-RDRP techniques that show a certain tolerance toward oxygen, light-mediated processes have greatly surged in recent years,^16–18^ including surface-initiated photoinduced atom transfer radical polymerization (SI-photoATRP) and surface-initiated photoinduced electron/energy transfer reversible addition–fragmentation chain-transfer (SI-PET-RAFT) polymerization. These versatile processes have been employed to fabricate a vast range of materials, including polymeric bioconjugates and 3D-printed nanostructured materials.^19,20^

In an analogous way to the corresponding solution process, SI-photoATRP is typically performed under UV light irradiation.^11,21,22^ During this process, ATRP deactivators XCu^II^L^+^ (X = Br or Cl, and L is a multidentate amine ligand) are photoexcited upon absorbing light and subsequently reduced by an electron donor—usually uncoordinated L present in an excess—to yield Cu^I^L^+^ activators, which establish the ATRP equilibrium in the presence of R-X initiator.^23–25^ Simultaneously, Cu^I^L^+^ species can coordinate O_2_ dissolved in the system, generating Cu^I^X­(O_2_)­L^+^; once photoexcited, these compounds can recombine with L, yielding oxidized species (L_ox_) while regenerating Cu^I^L^+^ activators.^26–28^

Within a closed or highly confined reaction setup that has not been deoxygenated (*i.e.*, with a limited amount of dissolved oxygen), this process continues until all O_2_ has been consumed. Oxygen consumption is mirrored by a certain induction time before steady polymer growth can be attained, whereas during SI-photoATRP within confined setups—such as in the case of surfaces covered by a transparent glass substrate^21^—continuous diffusion of oxygen from the open sides of the polymerization setup leads to inhibition of polymer brush growth at the “edges” of the substrate.

Hence, SI-photoATRP shows effective tolerance toward nondeoxygenated reaction mixtures just when highly confined polymerization setups are employed, *i.e.*, when oxygen diffusion toward the propagating brush interface is significantly hindered.^4,11,21,22^

Both the confinement and “edge effect” can be alleviated upon introducing an untethered ATRP initiator, resulting in the generation of radicals that quickly recombine with adventitious oxygen, limiting its detrimental effect on the polymerization.^4^ Nevertheless, the scalable fabrication of polymer brushes from diverse materials requires the development of fully open-air polymerization procedures, which are effective within industrially relevant settings, *i.e.*, without the application of confined polymerization setups.^3,5^

It was recently demonstrated that a fully oxygen-tolerant photoATRP can be attained by exploiting low-energy red/NIR-light irradiation in the presence of methylene blue (MB^+^) acting as a cocatalyst and dimethyl sulfoxide (DMSO), providing the controlled growth of chemically diverse polymers under open-air conditions.^29,30^

The mechanism of red-light-driven photoATRP involves a reductive quenching cycle whereby the excitation of MB^+^ generates the triplet-state ^3^MB^+^*, which is reduced by an electron donor to MB^•^(Figure 1). The latter reduces Cu^II^ deactivators by single electron transfer, generating Cu^I^L^+^ species and simultaneously reforming ground-state MB^+^.^29^ Effective consumption of O_2_ in the polymerization mixture is predominantly enabled through the reaction of ^3^O_2_ with ^3^MB^+^*, which forms singlet oxygen that reacts with DMSO to yield the corresponding sulfone (Figure 1).

**Figure 1:**
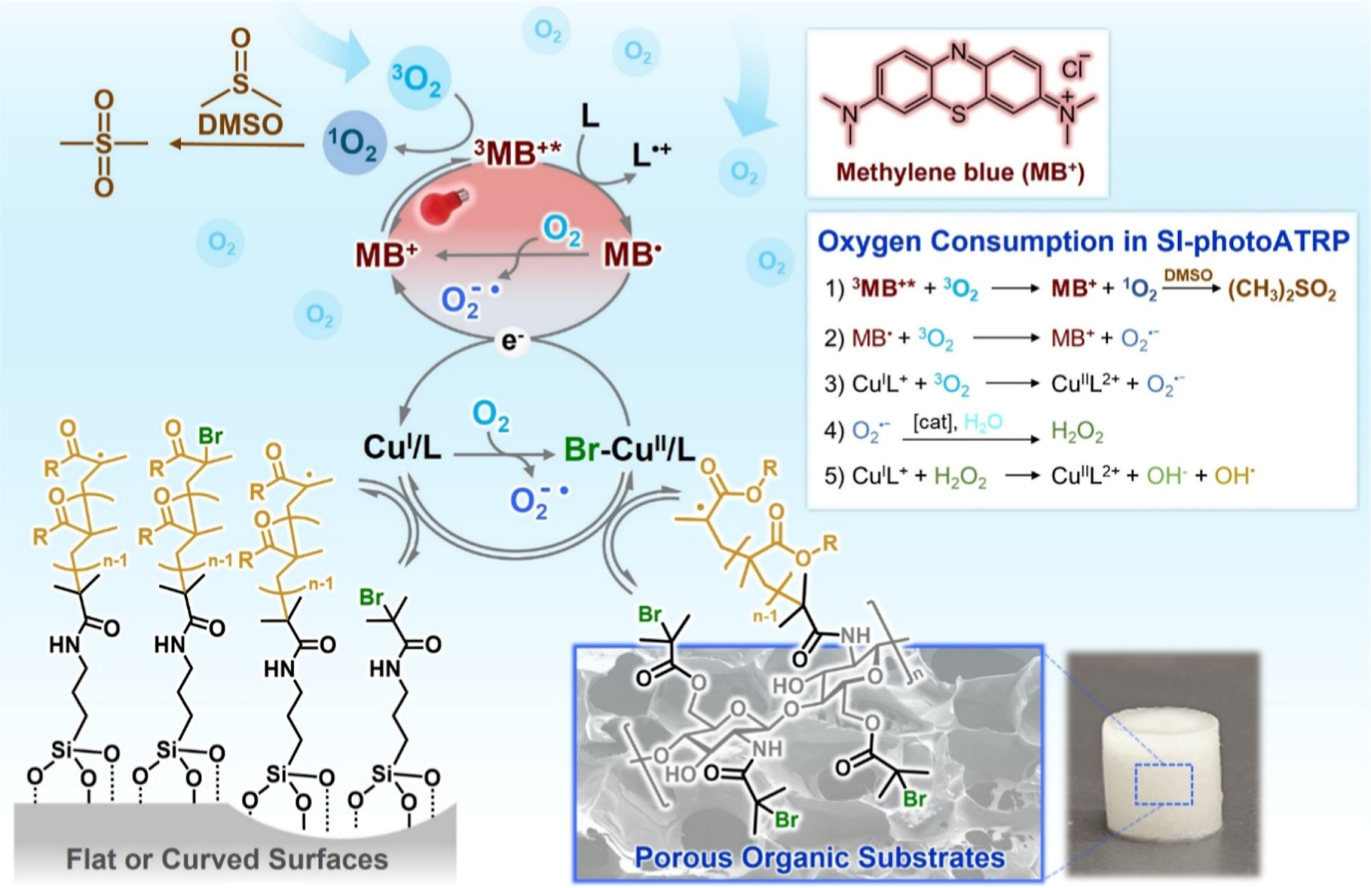
Proposed mechanism of SI-photoATRP in the presence of MB^+^ under red-light irradiation in open air.

The unprecedented tolerance of red-light-driven photoATRP toward environmental conditions makes it the ideal process for the fabrication of polymer brushes under technologically relevant settings. Red light features a high penetration depth within organic materials, enabling SI-photoATRP from three-dimensional (3D) supports and porous substrates. In addition, both red light and MB^+^ are biocompatible and harmless toward biological samples, potentially making red light-driven photoATRP an ideal tool for functionalizing morphologically different biomaterials.

Inspired by the attractive traits of the corresponding solution process, here we investigated the main features of red light-driven SI-photoATRP. We particularly focused on a catalytic system based on Cu/TPMA-MB^+^, which was activated through red-light irradiation at λ_max_ = 625 nm. The influence on brush growth by polymerization mixture composition, light intensity, and wavelength was systematically investigated. Optimization of grafting conditions enabled the fast growth of compositionally different brushes under completely open-air conditions, both from flat, macroscopic surfaces and from porous materials (Figure 1).

Especially within a microporous 3D material, we demonstrated that varying the wavelength of light that triggers ATRP equilibrium enables polymer grafting at different depths, alternatively stimulating brush growth just in its outer volume or uniformly across the support.

Red light-driven SI-photoATRP thus demonstrates an extremely versatile technique for generating brush coatings on a variety of substrates under open-air conditions, circumventing the requirements characterizing other oxygen-tolerant SI-RDRP methods.

## 2 Experimental Section

### 2.1 Functionalization of the SiO_*x*_ Substrate with an ATRP Initiator Layer

Silicon substrates were cut (1 cm × 2 cm) and cleaned with piranha solution (3:1 mixture (v/v) of H_2_SO_4_ and H_2_O_2_) for 1 h. Then, they were rinsed with ultrapure water and ethanol and dried under a stream of N_2_. The cleaned substrates were placed in a desiccator and functionalized with (3-aminopropyl)­triethoxysilane (APTES) by vapor deposition under vacuum. After 3 h, the functionalized substrates were washed with ultrapure water, ethanol, and toluene and then dried under a stream of N_2_. α-Bromoisobutyryl bromide (BiBB, 0.2 mL), triethylamine (TEA, 0.2 mL), and dry dichloromethane (DCM, 20 mL) were added to an Erlenmeyer flask containing the APTES-bearing substrates and let react for 2 h. Subsequently, the ATRP initiator-functionalized substrates were washed with DCM and ethanol and dried under N_2_.

### 2.2 Typical Procedure for the Preparation of the Polymerization Mixture

First, the following stock solutions were prepared: (i) 50 mM CuBr_2_, 300 mM TPMA, or Me_6_TREN in DMSO, and (ii) 25 mM MB^+^ in ultrapure water. A representative polymerization mixture was then prepared by mixing ultrapure water (3.45 mL), oligo­(ethylene glycol)­methyl ether methacrylate *M*_n_ ∼ 500 Da (OEGMA, 1 mL), DMSO (400 μL), an aliquot of the Cu complex stock solution (100 μL), and of the MB^+^ stock solution (50 μL). For some experiments, stock solutions of NaX (200 mM in milli-Q) and ethyl α-bromoisobutyrate (EBiB, 50 mM in DMSO) were prepared and added to the polymerization mixture as needed.

### 2.3 SI-PhotoATRP under Confinement

An initiator-functionalized substrate was placed on a glass Petri dish and covered with 1 μL cm^–2^ of a polymerization mixture and a glass slide. Then, the red-light (λ_max_ = 625 nm) source (CHROLIS C1 LED light source, Thorlabs) was turned on. The light source was placed at a distance of 15.5 cm from the substrate, and the light intensity measured on the substrate with a power meter (Thorlabs) was 4 mW cm^–2^. The intensity was varied by changing the distance of the light source from the substrate. After the desired polymerization time, the light was turned off, and the substrates were washed with ethanol and ultrapure water and dried under a stream of N_2_.

### 2.4 SI-PhotoATRP in Open Air

An initiator-functionalized substrate was placed on a glass Petri dish (with a diameter of 5.7 cm), which was filled with 5 mL of polymerization mixture uniformly covering the substrate. For some experiments, a smaller Petri dish (diameter of 2.9 cm) was employed to reduce the total reaction volume. The red-light source was positioned above the Petri dish at a distance of 25.5 cm to obtain a light intensity on the substrate of 4 mW cm^–2^. For SI-photoATRP under different light wavelengths, the same light source was employed and set to the desired wavelength (UV: λ_max_ = 365 nm, blue: λ_max_ = 420 nm, green/yellow: λ_max_ = 565 nm, NIR: λ_max_ = 780 nm). The distance of the source from the substrate was regulated to keep the light intensity on top of the substrate constant at 4 mW cm^–2^. After polymerization, the light was turned off, and the substrates were thoroughly washed with ultrapure water and ethanol, followed by drying under a stream of N_2_.

### 2.5 SI-PhotoATRP under Red Light in a Deoxygenated Environment

An initiator-functionalized substrate was placed vertically in a glass vial using Teflon spacers as a support. 3.5 mL of the polymerization mixture was added to the vial to cover the substrate, and it was degassed with Ar for 30 min. Then, the substrate was irradiated under the red-light source at a distance of 25.5 cm (light intensity of 4 mW cm^–2^). After the desired polymerization time, the light was turned off, and the vial stopper was removed. The substrate was washed with ultrapure water and ethanol and dried under a stream of N_2_.

### 2.6 Chitosan Cryogels Preparation and Functionalization with Polymer Brushes

Chitosan (CS) cryogels were synthesized following a reported procedure.^31^ First, 300 mg of CS was uniformly dispersed in 10 mL of ultrapure water. Then, 200 μL of acetic acid was added, and the mixture was stirred for 5 h at 40 °C. The resulting solution was transferred to a reaction mold and freeze-dried overnight to form a scaffold. Finally, the scaffold was immersed in a 2 M NaOH solution for 6 h to promote the cross-linking process.

The obtained CS cryogel was submerged in a solution composed of 10 mL of tetrahydrofuran (THF), 0.5 g of 4-dimethylaminopyridine (DMAP), and 0.5 mL of TEA. The solution was degassed with Ar for 30 min. Subsequently, 0.5 mL of BiBB in 10 mL of THF was added dropwise while the flask was maintained in an ice bath. After BiBB was added, the ice bath was removed, and the solution was stirred at room temperature for 24 h.

The ATRP initiator-functionalized CS cryogel was soaked with 0.4 mL of polymerization mixture comprising either OEGMA or 3-sulfopropyl methacrylate (SPMA) as the monomer (20 vol %, with 60 vol % water and 20 vol % DMSO), and irradiated for 4 h under either red or UV light (λ_max_ = 625 or 365 nm, respectively). After polymerization, the cryogel was thoroughly rinsed with ultrapure water and ethanol and freeze-dried.

### 2.7 SI-PhotoATRP in Open Air from Stainless Steel Objects

Stainless steel objects were cleaned and functionalized with ATRP initiators using similar protocols to those for SiO_*x*_ substrates (Section 2.1). The functionalized blade was placed on a Petri dish and covered with 5 mL of polymerization mixture. The Petri dish was placed under a red-light source at a light intensity of 4 mW cm^–2^ for 4 h. In the case of the spoon, the polymerization mixture was dropped inside the initiator-functionalized spoon bowl, which was irradiated with a red-light source at a light intensity of 4 mW cm^–2^ for 10 h. After polymerization, the objects were washed with ultrapure water and ethanol and dried under a stream of N_2_.

## 3 Results and Discussion

### 3.1 SI-PhotoATRP under Red Light in a Confined Setup

SI-photoATRP in the presence of MB^+^ and under red-light irradiation was initially investigated within confined environments by dispensing a nondegassed polymerization mixture (1 μL cm^–2^) on ATRP initiator-functionalized SiO_*x*_ substrates and subsequently covering the surface with a borosilicate glass slide (Figure 2a,b). Polymerization solutions comprised OEGMA (20 vol %), H_2_O (70 vol %), DMSO (10 vol %), CuBr_2_/TPMA as the catalyst and MB^+^ as the cocatalyst ([MB^+^]/[Cu] = 0.25), and a 5-fold excess of TPMA relative to Cu to serve as the electron donor. Following 30 or 60 min of red-light irradiation (λ_max_ = 625 nm) at an intensity of 35 mW cm^–2^, POEGMA brushes with dry thicknesses (*T*_dry_) of 16 ± 1 and 20.3 ± 0.4 nm, respectively, were successfully fabricated, as confirmed by variable angle spectroscopic ellipsometry (VASE) (Figures 2c in S1). When irradiation was prolonged until 180 min, relatively thicker POEGMA brushes were generated, reaching *T*_dry_ = 27 ± 2 nm. By varying the composition of the polymerization mixture, the role of each component could be highlighted. As shown in Table S1 (entries 1–4), both CuBr_2_ and TPMA were necessary to obtain substantial brush growth. Either lower or higher concentrations of CuBr_2_ resulted in thinner POEGMA brushes (*i.e.*, lower molar mass), whereas increasing [MB^+^] had no substantial effect on the film thickness (Table S1, entries 5–8).

**Figure 2:**
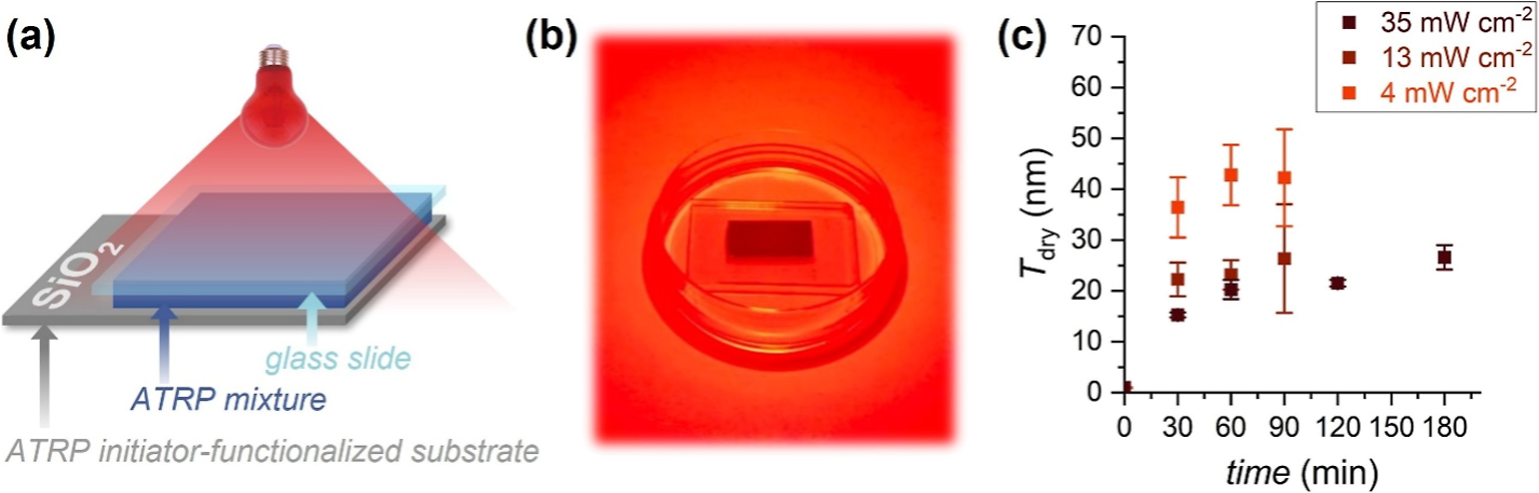
SI-photoATRP of OEGMA under red-light irradiation (λ_max_ = 625 nm) in a confined setup: (a) schematics and (b) image of the employed setup; (c) dry thickness of POEGMA brushes with varying the intensity of red-light irradiation and the reaction time. Polymerization conditions: OEGMA 20 vol %, DMSO 10 vol %, H_2_O 70 vol %, 1 mM CuBr_2_, [MB^+^]:[CuBr_2_]:[TPMA] = 0.25:1:6.

In contrast, the intensity of red light played a major role in the polymerization process. Relevantly, when the light intensity was decreased to 13 and 4 mW cm^–2^, *T*_dry_ increased to 22.3 ± 3.3 and 36.4 ± 5.9 nm, respectively, after 30 min of irradiation (Figure 2c). An increase in light intensity was presumably mirrored by a concomitant increment in the concentration of the Cu^I^ activator within the polymerization mixture. Hence, as it was previously observed for zerovalent metal-mediated SI-ATRP, higher concentrations of Cu^I^ activator did not translate into faster brush growth but rather led to an increment in the occurrence of chain termination and thus a decrease in brush thickness and/or grafting density.^7,32,33^ Notably, a steady growth of brushes was attained by a 6-fold decrease of light intensity compared to what was previously recorded for polymerizations performed in solution (4 mW cm^–2^ for SI-photoATRP vs 25 mW cm^–2^ for photoATRP in solution).^29,30^

### 3.2 SI-PhotoATRP under Red Light in Open Air

While growing polymer brushes within highly confined environments enables the consumption of oxygen dissolved in the mixture and thus avoids deoxygenation, the possibility of growing polymer brushes in completely open air could greatly simplify the reaction setup, in this way broadening the substrate scope and the applicability of the entire process. The addition of MB^+^ in photoATRP introduces various routes for O_2_ scavenging, as highlighted in Figure 1.

These include (i) the reaction of excited ^3^MB* with ^3^O_2_, (ii) the oxidation of MB^•^, and (iii) the oxidation of Cu^I^ species generated through the reductive quenching cycle (Figure 1).^29^ Therefore, we attempted to grow POEGMA brushes from an ATRP initiator-functionalized SiO_*x*_ substrate placed in the middle of an uncovered Petri dish (Figure 3a). The Petri dish was filled with 5 mL of a reaction mixture to submerge the substrate. Upon 90 min of red-light irradiation at an intensity of 4 mW cm^–2^, POEGMA brush films with a *T*_dry_ = 146 ± 7 nm were obtained (Table 1, entry 1). Control experiments indicated that both MB^+^ and CuBr_2_/TPMA were needed to ensure the growth of POEGMA brushes (Table 1, entries 2 and 3).

**Figure 3:**
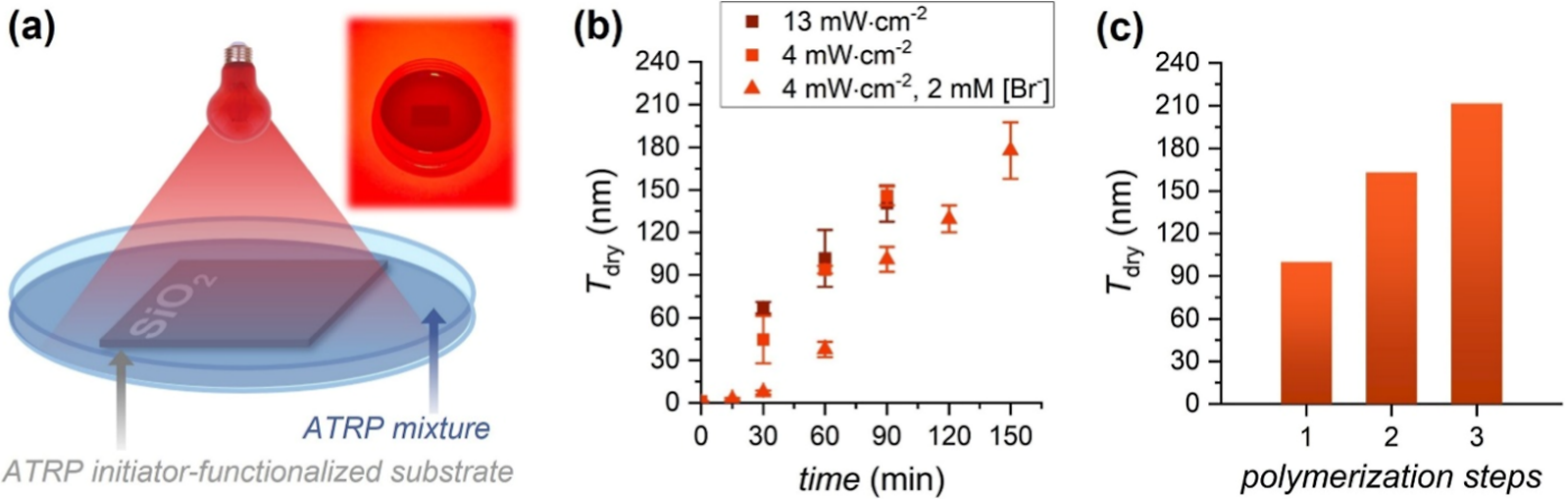
SI-photoATRP of OEGMA under red-light irradiation (λ_max_ = 625 nm) in open air. (a) Schematized experimental setup with the corresponding picture. (b) Polymer brush growth rates recorded by ex situ VASE, following SI-photoATRP performed by irradiating the polymerization setup with red light at different intensities and with/without the addition of NaBr. (c) Dry thickness of POEGMA brushes measured after 1 h of irradiation for 3 consecutive polymerization steps on the same substrate. Conditions: OEGMA 20 vol %, DMSO 10 vol %, H_2_O 70 vol %; 1 mM CuBr_2_, [MB^+^]:[CuBr_2_]:[L] = 0.25:1:6; λ = 625 nm, and light intensity = 4 mW cm^–2^, unless otherwise stated.

**Table 1:** Open Air SI-PhotoATRP of OEGMA under Red-Light Irradiation^a^

entry	[MB^+^] (mM)	[CuBr_2_] (mM)	[TPMA] (mM)	[NaBr] (mM)	*T*_dry_ (nm)^b^
1	0.25	1	6		146 ± 7
2	0.25		6		1.1 ± 0.1
3	0.25	1			1.1 ± 0.1
4^c^	0.25	1	6		140 ± 13
5^d^	0.25	1	6		113 ± 1
6	0.1	1	6		118 ± 13
7	0.05	1	6		119 ± 10
8	0.125	0.5	3		134 ± 8
9	0.25	1	6	5	93 ± 4
10	0.25	1	6	2	106 ± 1
11^e^	0.25	1	6	2	95 ± 1

a Polymerization conditions: OEGMA 20 vol %, DMSO 10 vol %, H_2_O 70 vol %; irradiated for 90 min under red light (λ_max_ = 625 nm, 4 mW cm^–2^).

b Measured by VASE.

c Light intensity = 13 mW cm^–2^.

d In an Ar-degassed, closed system.

e DMSO 20 vol %, H_2_O 60 vol %.

Upon decreasing [MB^+^] to 0.1 and 0.05 mM, the resulting brush films exhibited lower *T*_dry_ values of ∼ 120 nm. Additionally, a slight decrease in *T*_dry_ to ∼ 134 nm was recorded upon reducing [CuBr_2_] to 0.5 mM while keeping constant [MB^+^]/[Cu] and [Cu]/[L] (Table 1, entries 6–8).

The use of 10 vol % DMSO was essential to enable SI-photoATRP to occur in an open-air setup. Indeed, no brush growth was measured in the absence of DMSO, whereas a relatively thin, highly inhomogeneous film was obtained by performing SI-photoATRP with only 2 vol % of DMSO (Figure S2). In contrast, DMSO contents higher than 10 vol % did not translate into a substantial benefit, while using the sole DMSO as solvent decreased the polymerization rate, generating brushes with *T*_dry_ = 44 ± 3 nm after 3 h of irradiation (Table S2). Under these conditions, the observed slower brush growth could be attributed to the lower polarity of DMSO relative to a mixture of water/DMSO (7/1 v/v), which reduced the equilibrium constant and the rate constant of activation of dormant species in ATRP.^34^

Under optimized conditions, brush growth rates measured by ex situ VASE revealed a linear increase in polymer brush thickness over time (Figure 3b) with no evident induction period. This behavior suggests a controlled polymerization process^1^ in which oxygen is readily scavenged and cannot hamper the radical generation and propagation processes. Relevantly, the application of a higher light intensity of 13 mW cm^–2^ had no substantial effect on the brush growth kinetics (Figure 3b and Table 1, entry 4), whereas the brush films obtained under these conditions were less uniform, presumably due to chain termination reactions caused by an increase in activator contents (Figure S3).

Sequential polymerization/chain extension steps allowed us to generate brushes with high thicknesses. A first SI-photoATRP step led to POEGMA brushes with *T*_dry_ of 100 ± 5 following 1 h of irradiation. After extensive rinsing, the substrate was placed in a newly prepared reaction mixture and irradiated for an additional 1 h, leading to a marked increase in the POEGMA brush thickness, which reached *T*_dry_ = 163 ± 8 nm. A third polymerization step finally resulted in a *T*_dry_ of 212 ± 14 nm. The progressive and rather linear increase in brush thickness upon sequential polymerization/chain extension stages (Figure 3c) suggests that red light-mediated SI-photoATRP was well-controlled and confirms the suitability of this method for synthesizing high-molar mass brushes (*i.e.*, significantly thick films with *T*_dry_ > 200 nm).^35^

It is important to emphasize that by applying an irradiation time *t* ≥ 60 min, a non-negligible amount of polymer was generated in solution, especially concentrated near the substrate, *i.e.*, in the proximity of the brush propagating front. For *t* > 90 min, the polymer layer was very difficult to remove from the substrate. Sampling of the polymerization mixture after 90 min of light irradiation allowed us to estimate a monomer conversion of ∼ 50% (as measured by ^1^H NMR) in the reaction volumes nearby the substrate, whereas monomer conversion <10% was recorded in the bulk solution (further away from the substrate).

A possible cause of radical generation in solution stems from the reaction of dissolved O_2_ with either MB^•^or Cu^I^L^+^, resulting in the formation of the superoxide anion O_2_^•‑^ (Figure 1). The latter can be further reduced to H_2_O_2_ ^36,37^ that reacts with Cu^I^L^+^ via a Fenton-type reaction to form hydroxyl radicals, HO^•^, which can initiate new chains. These oxygen-scavenging pathways were generally negligible for similar photoATRP systems in solution; however, their contribution became more pronounced upon decreasing the concentration of ATRP initiator below 0.3 mM (*i.e.*, targeting a degree of polymerization of 1000 or higher).^29^ Considering that in SI-photoATRP, the concentration of surface-bound initiator is extremely small (as it is in the case of any surface-confined ATRP process), it is plausible that the generation of O_2_^•‑^ and the consequent formation of propagating radicals in solution cannot be neglected.

While these radicals can cause polymerization to occur in solution, they can also contribute to shifting the ATRP equilibrium to the Cu^I^ activator, thus accelerating surface polymerization. This phenomenon could contribute to the higher film thickness obtained in open air in comparison to the confined system under similar reaction conditions (*i.e.*, *T*_dry_ = 42 ± 10 nm vs 146 ± 7 nm upon 90 min of reaction with red-light irradiation at 4 mW cm^–2^, Figures 2c and 3b, respectively).

It is also relevant to highlight that a similar SI-photoATRP process performed within a sealed vial by employing 3.5 mL of carefully deoxygenated polymerization mixture resulted in POEGMA brushes with *T*_dry_ = 113 ± 1 nm after 90 min (*i.e.*, ∼ 20% lower thickness compared to that obtained through an open air process) with no signs of polymer formed in solution. This further indicates that O_2_ plays a role in radical generation in solution.

We attempted to facilitate the removal of unbound polymer from the substrate by limiting its molecular weight through the addition of an untethered ATRP initiator, EBiB, to the polymerization mixture. Simultaneously, EBiB can enhance O_2_ consumption by generating radicals that can recombine with oxygen. However, the polymerization from the surface became progressively slower with an increase in [EBiB] (Table S3). The observed slower polymer brush growth can be attributed to the accumulation of Cu^II^-based deactivators, caused by the termination of radical species generated in solution from EBiB upon reaction with O_2_ and derived species.^4^ Additionally, size exclusion chromatography (SEC) analysis of the polymerization mixture collected after 90 min of SI-photoATRP in the presence of 0.1 mM EBiB showed a polymer with *M*_n_ values of ∼ 135 kDa and D̵ values of ∼ 2.8 (Figure S4). Since a polymer layer concentrated on top of the substrate was visible, we attempted to analyze this unbound polymer by GPC, which revealed an *M*_n_ of ∼ 174 kDa and a very broad molecular weight distribution (D̵ ∼ 14, Figure S4). Thus, radical generation and uncontrolled propagation still occur in the presence of a free initiator.

Nevertheless, it must be noted that no polymer in solution could be visually detected when investigating monomers different from those of the OEGMA (*vide infra*). In addition, the extent of polymerization in solution was dependent on the height of the solution film above the substrate and on the ratio of substrate area to reaction volume (*S*/*V*). When the *S*/*V* was increased from 0.4 to 1.6 cm^–1^ (Table S4), the substrate could be easily cleaned after 90 min of polymerization, which returned a brush thickness of 107.9 ± 2.0. We hypothesized that a substantial increase in *S*/*V* would reduce the exchange surface available for the O_2_ molecules, mitigating its impact on the reactions. The increase in *S*/*V* was obtained by reducing both the size of the Petri dish and the reaction volume. These parameters influenced not only the extent of solution polymerization but also the brush thickness (Table S4). Indeed, thicker polymer brush films were obtained upon reduction of the height of the liquid mixture covering the substrate. Various factors can contribute to this trend, including O_2_ diffusion and light penetration through the polymerization mixture. We can thus conclude that the optimal setup for growing brushes in open-air systems features (i) high *S*/*V* and (ii) the lowest amount of polymerization mixture that offers a uniform coverage of the substrate.

Finally, the effect of halide salts on red-light-mediated SI-photoATRP in open air and *S*/*V* = 0.4 cm^–1^ was investigated. Aqueous ATRP systems are characterized by high values of the ATRP equilibrium and activation rate constants, *K*_ATRP_ and *k*_act_, respectively, and low stability of the deactivator X-Cu^II^L^+^, which tends to dissociate into X^–^ and Cu^II^L^2+^ (which cannot deactivate propagating radicals).^38,39^ Hence, a large excess of halide ions relative to the Cu catalyst is generally introduced to increase the deactivation efficiency and promote control over the polymer growth. For aqueous SI-ATRP, controlled brush growth has been reported both in the absence and presence of halide salts.^40–45^

The addition of NaBr or NaCl to open air SI-photoATRP caused a decrease in the POEGMA brush growth rate (Figure 3b and Table S5), with average film thickness decreasing with increasing [NaX]. For [NaX] = 5 mM, *T*_dry_ = 40 ± 4 and 93 ± 4 nm were measured for X = Cl and X = Br, respectively, after 90 min of irradiation. This result indicated that the addition of NaX improved deactivator stability—with Cl^–^ binding more strongly to Cu^II^ complexes than Br^39^—and promoted deactivation efficiency, consequently slowing polymer brush growth.

Simultaneously, the introduction of NaX sensibly decreased the extent of polymer formation in solution. In the case of reaction mixtures comprising [NaBr] = 2 mM polymerization/irradiation time could be extended to 150 min while avoiding the formation of a significant amount of polymer in solution and obtaining POEGMA brushes with *T*_dry_ = 178 ± 20 nm. Under these conditions, the increase in brush thickness remained linear over time; however, a short induction period of ∼ 15 min was recorded. This could be attributed to the increased concentration of effective deactivator, which determined a longer irradiation time necessary to generate a sufficient amount of Cu^I^ activating species.

### 3.3 Monomer Scope and Irradiation Wavelengths

The monomer scope for red light-mediated SI-photoATRP in open air was tested employing different water-soluble monomers (Table 2). Polymer brushes were effectively grown using neutral, zwitterionic, and negatively charged methacrylate monomers (Figure S5), including OEGMA of various OEG side-chain lengths, 2-methacryloyloxyethyl phosphorylcholine (MPC), 2-(*N*-3-sulfopropyl-*N*,*N*-dimethylammonium)­ethyl methacrylate (DMAPS), and 3-sulfopropyl methacrylate (SPMA). The polymerization of oligo­(ethylene glycol)­methyl ether acrylate (OEGA) was markedly slower than that of the corresponding methacrylate monomers, even by employing a more active ATRP catalyst, *i.e.*, Cu/tris­[2-(dimethylamino)­ethyl]­amine (Me_6_TREN). In contrast, poly­(*N*-isopropylacrylamide) (PNIPAM) brushes were grown more rapidly by employing Cu/Me_6_TREN than with Cu/TPMA, reaching *T*_dry_ = 89 ± 8 and 3 ± 1 nm, respectively, after 90 min of irradiation.

**Table 2:** Open Air Red Light-Mediated SI-PhotoATRP of Different Monomers^a^

entry	monomer (M)	ligand (L)	*T*_dry_ (nm)^b^
1	OEGMA (*M*_n_ ∼ 500 g mol^–1^)^c^	TPMA	106 ± 1
2	OEGMA (*M*_n_ ∼ 950 g mol^–1^)	TPMA	10 ± 1
3	NIPAM	TPMA	3 ± 1
4	NIPAM	Me_6_TREN	89 ± 8
5	OEGA^c^	Me_6_TREN	5 ± 1
6	MPC	Me_6_TREN	21 ± 6
7	DMAPS	Me_6_TREN	5 ± 1
8	SPMA	Me_6_TREN	13 ± 1

a Conditions: Monomer 20 vol %, DMSO 10 vol %, H_2_O 70 vol %; 1 mM CuBr_2_, [MB^+^]:[CuBr_2_]:[L] = 0.25:1:6; irradiated for 90 min under red light (λ_max_ = 625 nm, 4 mW cm^–2^).

b Measured by VASE.

c [MB^+^]:[CuBr_2_]:[L]:[NaBr] = 0.25:1:6:2.

Additionally, MB^+^ has a rather broad absorption spectrum (Figure 4); thus, the Cu/L-MB^+^ dual catalytic system could be used to catalyze photoATRP under irradiation at different light wavelengths.^29^ POEGMA brush films with *T*_dry_ ∼ 95 nm (Figure 4 and Table S6) were obtained upon 90 min exposure to either UV or green/yellow light (λ_max_ = 365 and 565 nm, respectively, at an intensity of 4 mW cm^–2^). In contrast, no brush growth was observed by subjecting the polymerization setup to blue (λ_max_ = 420 nm) and NIR (λ_max_ = 780 nm) light irradiation at the same intensity due to the minimal absorbance of MB^+^ in these spectral regions (Figure 4).

**Figure 4:**
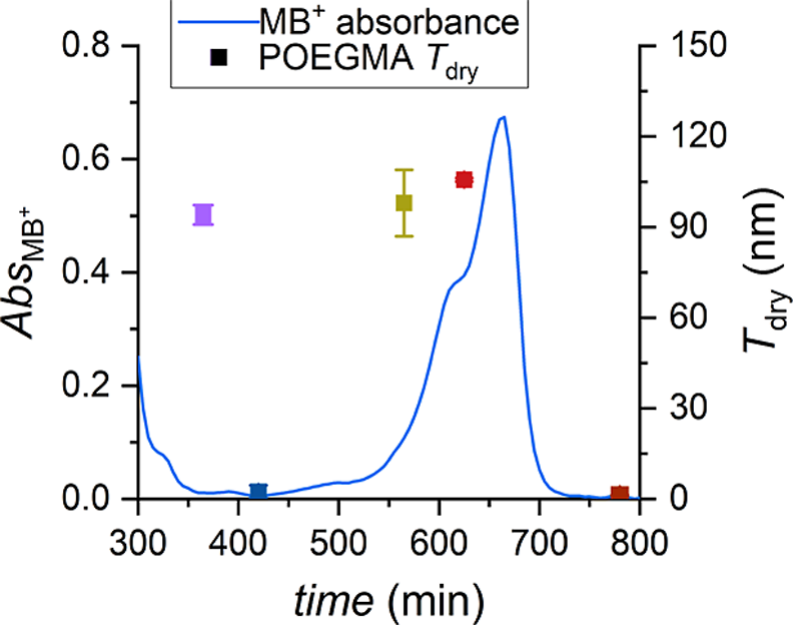
UV–vis spectrum of MB^+^ (blue line) and dry thickness of POEGMA brushes as a function of the applied light wavelength (squares). Conditions: OEGMA 20 vol %, DMSO 10 vol %, H_2_O 70 vol %; 1 mM CuBr_2_, [MB^+^]:[CuBr_2_]:[L]:[NaBr] = 0.25:1:6:2; irradiated for 90 min under a light intensity of 4 mW cm^–2^.

### 3.4 SI-PhotoATRP from 3D Substrates

An important advantage of low-energy light over shorter wavelengths is represented by its enhanced penetration through non-transparent materials. For instance, Hawker, Boyer et al. exploited a NIR light-mediated SI-PET-RAFT polymerization to grow polymer brushes within the inner walls of opaque silicone tubes.^12^ Thus, the enhanced penetration offered by the red light-mediated SI-photoATRP could enable the growth of polymer brushes within porous substrates, uniformly decorating not only the external walls but also the interior of pores, cavities, and channels. To demonstrate this, the Cu/L-MB^+^ catalytic system was employed to functionalize a CS-based cryogel (Figures 1 and S6) in open air. 3D substrates based on CS are technologically relevant supports for polymer brush modification, as they have been frequently employed as tissue engineering constructs and, more generally, as biocompatible biomaterial platforms.^31,46,47^

CS cryogels were reacted with BiBB to introduce C–Br groups that served as initiating sites for the subsequent SI-photoATRP. The modified supports were later soaked in a polymerization mixture comprising MB^+^, CuBr_2_/TPMA, and SPMA or OEGMA as monomer in water with 10 vol % DMSO. Soaked supports were irradiated for 90 min with red light. After extensive washing, the 3D supports were sectioned and analyzed by attenuated total reflectance infrared spectroscopy (ATR-IR) and scanning electron microscopy coupled with energy-dispersive X-ray spectroscopy (SEM-EDS) in order to characterize the composition of both the inner volumes and the outer part of the porous structure (Figure 5a–c). Following red-light irradiation, a uniform growth of polymer brushes was recorded across the entire CS structure in the case of both PSPMA and POEGMA (Figures 5b, S7a, and S8). When a similar grafting process was repeated on a new set of initiator-functionalized supports under UV light irradiation (λ_max_ = 365 nm, 4 mW cm^–2^), the growth of brushes was evidently more marked on the external volumes of the gel structure, while ATR-IR recorded little or no evidence of polymer grafts within the inner section of the cryogels (Figures 5c, S7b, and S8). Hence, the enhanced penetration of red light enabled homogeneous functionalization of 3D porous materials in an open-air setup. At the same time, SI-photoATRP exploiting Cu/L-MB^+^ catalytic systems demonstrated an extremely powerful and versatile technique to decorate 3D materials with functionalization depths that could be tuned by varying light irradiation wavelength.

**Figure 5:**
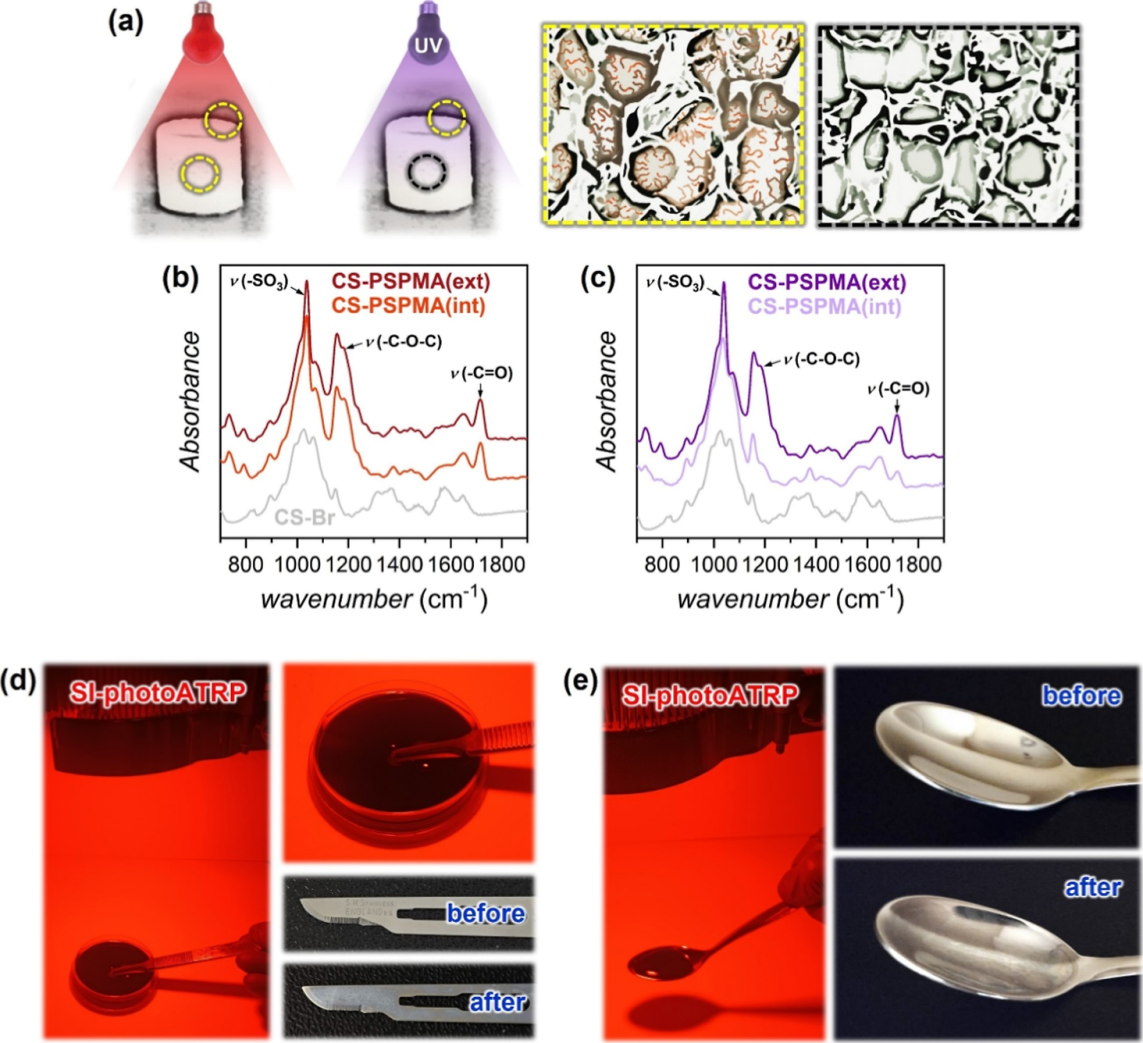
(a) SI-photoATRP was applied in open air to grow polymer brushes from a 3D CS cryogel previously functionalized with the ATRP initiator: when UV light with limited penetration was employed, brush growth only occurred on the external surface of the material. In contrast, uniform functionalization throughout the material, including its pores, was achieved under red-light irradiation. (b) ATR-IR spectra showing uniform growth of PSPMA brushes inside the pores (interior, “int”) and on the outer surface (exterior, “ext”) of functional CS cryogels, by employing red light. (c) ATR-IR spectra showing prevalent growth of PSPMA brushes on the outer surface (ext) of functional CS cryogels by employing UV light. (d,e) Functionalization of stainless-steel objects with POEGMA brushes in open air.

Finally, to demonstrate the versatility of red-light-mediated SI-photoATRP, we focused on functionalizing objects with varying shapes and surface curvatures, such as a stainless-steel blade and a spoon (Figure 5d,e). ATRP initiators were deposited on the surfaces of these objects using an approach similar to that employed for SiO_2_ substrates. The blade was placed in a Petri dish, similar to the flat SiO_2_ substrates, covered with the standard polymerization mixture, and irradiated with red light for 4 h. To grow brushes from the curved surface of the spoon, the polymerization mixture was poured directly into the spoon, which was then irradiated with red light from above. After thorough washing, both objects showed visibly less reflective surfaces, and the change in color reflected the presence of a uniform and relatively thick brush coating.

Red light-driven SI-photoATRP in open air gives access to growing polymer brushes at scale from materials with diverse shapes, overcoming the limitations imposed by confined setups and facilitating the implementation of SI-ATRP in realistic settings.

## 4 Conclusions

The combination of MB^+^- and Cu-based ATRP catalyst enables red light-driven SI-photoATRP under fully open-air conditions, addressing long-standing limitations related to oxygen sensitivity and confinement requirements in traditional SI-ATRP methods. Rapid growth of polymer brushes with tunable dry thickness was achieved from flat substrates, using different monomers and water as the solvent, with a small fraction of DMSO that contributes to scavenging oxygen.

The high penetration depth of red light within organic materials was exploited to uniformly grow polymer brushes from 3D microporous supports. Additionally, the possibility of activating the MB^+^-Cu/L catalytic system with different irradiation wavelengths enabled the selective growth of brushes from the support in different positions, *i.e.*, throughout its entire structure (using red light) or mainly at its outer surface (exploiting UV light).

Finally, this approach enabled the growth of brushes on objects with complex geometries, including curved and irregular surfaces, demonstrating versatility and scalability that are unattainable with traditional SI-ATRP systems.

Red light-mediated SI-photoATRP emerges as a robust, fully oxygen-tolerant process that can be translated into relevant industrial and biomedical settings, where process simplicity, material diversity, and scalability are essential.

## Supplementary Material



## Notes

Materials and instruments, additional data, and substrate photographs (PDF)
