# Supplementary material for: Open-Air Growth of Polymer Brushes by Surface-Initiated PhotoATRP under Red-Light Irradiation
Source: ACS Appl Mater Interfaces. 2025 Jun 18;17(26):38773–82. doi: 10.1021/acsami.5c08584 (PMC12232278; doi:10.1021/acsami.5c08584)
Supplement: Supplementary file 1 [file am5c08584_si_001.pdf]

SUPPORTIN INFORMATION

Open-Air Growth of Polymer Brushes by Surface-Initiated PhotoATRP under Red-Light Irradiation

Yuwen Zhang[1], Alessio Lo Bocchiaro[1], Xiaolei Hu[2], Carlos Pavon[1], Cristian Pezzato[1], Krzysztof Matyjaszewski[2], Francesca Lorandi[1]*, Edmondo M. Benetti[1]**

[1]Laboratory for Macromolecular and Organic Chemistry, Department of Chemical Sciences, University of Padova, via Marzolo 1, 35131 Padova, Italy.

[2]Department of Chemistry, Carnegie Mellon University, 4400 Fifth Avenue, Pittsburgh, Pennsylvania 15213, United States.

Emails: km3b@andrew.cmu.edu; francesca.lorandi@unipd.it; edmondo.benetti@unipd.it

S1. Materials

Ethanol (absolute for analysis, Supelco), ultrapure water (Millipore Milli-Q grade), anhydrous dichloromethane (DCM, ≥99.8%, Sigma-Aldrich), DCM (≥99.9%, Sigma-Aldrich), toluene (≥99.7%, Sigma-Aldrich), tetrahydrofuran (THF, ≥99.9%, Sigma-Aldrich) anhydrous dimethyl sulfoxide (DMSO, ≥99.9%, Sigma-Aldrich), sulfuric acid (H_2SO_4, 95-97%, Sigma-Aldrich), hydrogen peroxide (H_2O_2, 35 wt% in water, TCI), phosphate-buffered saline (PBS, Sigma-Aldrich), acetic acid (99.8%-100.5%, Sigma-Aldrich), (3-aminopropyl)triethoxysilane (APTES, ⩾98%, Sigma-Aldrich), α-bromoisobutyryl bromide (BiBB, 98%, Sigma-Aldrich), ethyl α-bromoisobutyrate (EBiB, 98%, Sigma-Aldrich), triethylamine (TEA, ≥99.5%, Sigma-Aldrich), methylene blue hydrate (MB^+, ≥95%, Sigma-Aldrich), tris(2-pyridylmethyl)amine (TPMA, 97%, Ambeed), tris[2-(dimethylamino)ethyl]amine (Me_6TREN, 97%, Sigma-Aldrich), copper(II) bromide ($CuBr_2$, 99%, Sigma-Aldrich), sodium chloride (NaCl, 99%, Sigma-Aldrich), sodium bromide (NaBr, 99%, Sigma-Aldrich), 4-dimethylaminopyridine (DMAP, ⩾99%, Sigma-Aldrich), and chitosan (CS, low molecular weight, Sigma-Aldrich) were used as received.

Oligo(ethylene glycol) methyl ether methacrylate (OEGMA, M_n ~500 Da, Sigma-Aldrich), oligo(ethylene glycol) methyl ether acrylate (OEGA, M_n ~480 Da, Sigma-Aldrich), 3-sulfopropyl methacrylate (SPMA, 98%, Sigma-Aldrich), 2-(*N*-3-sulfopropyl-*N*,*N*-dimethyl ammonium)ethyl methacrylate (DMAPS, 95%, Sigma-Aldrich), 2-methacryloyloxyethyl phosphorylcholine (MPC, 97%, Sigma-Aldrich), N-isopropylacrylamide (NIPAM, >98%, TCI), were passed through a column of aluminum oxide (activated, basic, Sigma-Aldrich) to remove the inhibitors. OEGMA, M_n ~950 Da (Sigma-Aldrich) was used as received.

Silicon wafers were purchased from Si-Mat (Landsberg, Germany).

S2. Instruments

Variable-angle spectroscopic ellipsometry (VASE). The dry thickness of polymer brushes (T_{dry}) was measured by using a M-2000V variable-angle spectroscopic ellipsometer (VASE, J.A. Woollam Co.) equipped with a 50 W Quartz Tungsten Halogen lamp (FQTH-100). Amplitude (Ψ) and phase (Δ) were acquired at an angle of incidence of 65° as a function of wavelength (370–1000 nm). The fitting of raw data was analyzed using WVASE32 software, employing a layered

model with bulk dielectric functions for Si and SiO_2. The polymer brush layers were characterized using a Cauchy model defined as $n=A+B\lambda^{-2}$, where A and B were set to 1.45 and 0.01 (values for transparent organic films). Here, λ is the wavelength, and n represents the refractive index. The thickness of each sample was measured on three spots to calculate the average T_{dry} values and standard deviations.

Attenuated total reflectance infrared (ATR-IR) spectroscopy. The structure of chitosan cryogels prior and after functionalization were analyzed by an ATR-IR spectrometer Cary 630 with a diamond ATR crystal. The spectra were recorded in the range from 4000 cm^{-1} to 600 cm^{-1}, and 128 scans at a resolution of 4 cm^{-1}.

Scanning electron microscopy and energy-dispersive X-ray spectroscopy (SEM-EDS). The morphology and composition of chitosan cryogels before and after functionalization were characterized by SEM through a FEI Quanta 200 (Netherlands) scanning electron microscope equipped with energy dispersive X-ray spectroscopy (EDS, Oxford Xplore 30, USA).

Nuclear magnetic resonance (NMR) spectroscopy. ^{1}HNMR spectra were recorded using Bruker Advanced III 400 MHz spectrometers at room temperature and using D_2O as solvent.

Gel permeation chromatography (GPC). SEC was performed by using a Viscotek gel permeation chromatography (GPC) system (Malvern, Worcs, U.K.) equipped with a pump and degasser (GPCmax VE2001, 1.0 mL min^{-1} flow rate), a detector module (Viscotek 302 TDA), and two columns (2× PLGel Mix-C, dimensions 8 mm × 300 mm for each column) using DMF with 10 mM LiBr as eluent. Each sample was prepared by dissolving the polymer at a defined concentration of 1 mg mL^{-1} in DMF containing 10 mM LiBr. Poly(methyl methacrylate) standards with molecular weight ranging from 2500 to 212000 Da were used for calibration.

S3. Additional data for SI-photoATRP under red light in a confined space

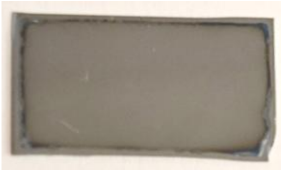

Figure S1. Photograph of the SiO_2 substrate after SI-photoATRP under red light in a confined setup. Conditions: OEGMA 20 vol%, DMSO 10 vol%, H_2O 70 vol%, 1 mM $CuBr_2$, $[MB^+]$:$[CuBr_2]$:[TPMA] = 0.25:1:6, red light irradiation (λ_{max} = 625 nm, 35 mW cm^{-2}) for 60 min.

Table S1. SI-photoATRP of OEGMA under red light irradiation, in a confined setup.[a]

Entry	$[MB^+]$ (mM)	$[CuBr_2]$ (mM)	[TPMA] (mM)	T_{dry} (nm)[b]
1	0.25	0	0	2.9 ± 0.1
2	0.25	0	6	3.9 ± 0.1
3	0.25	1	0	1.8 ± 0.1
4	0.25	1	6	20.3 ± 0.4
5	0.125	0.5	3	12.3 ± 0.7
6	1.25	5	30	16.2 ± 2.0
7	0.25	0.5	6	11.5 ± 0.9
8	1	1	6	20.9 ± 0.5

[a]Conditions: OEGMA 20 vol%, DMSO 10 vol%, H_2O 70 vol%; red light irradiation (λ_{max} = 625 nm, 35 mW cm^{-2}) for 60 min. [b]Measured by VASE.

S4. Additional data for SI-photoATRP under red light in open air

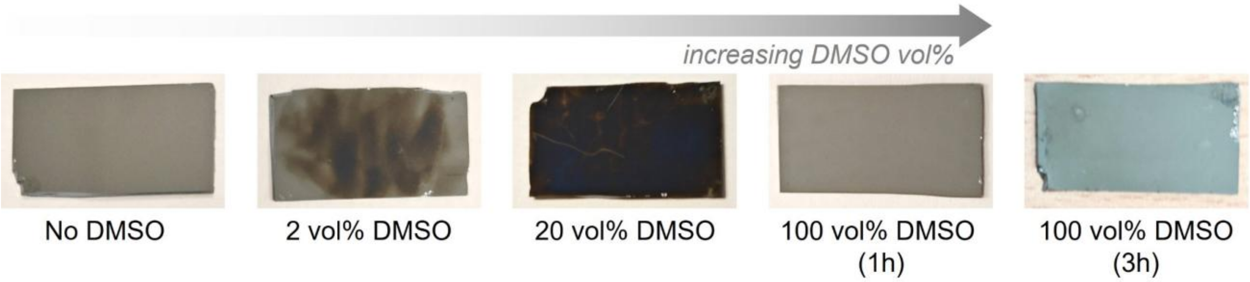

Figure S2. Photographs of the substrates after SI-photoATRP under red light in open air with varying solvent composition. The polymerization time was 1 h unless otherwise indicated. Polymerization conditions and brush thicknesses are reported in **Table S2** below.

Table S2. Open-air SI-photoATRP of OEGMA under red light irradiation with different solvent composition.[a]

**Entry**	**H_2O (vol%)**	**DMSO (vol%)**	**time (min)**	**T_{dry} (nm)[b]**
1	80	-	60	-
2	78	2	60	inhomogeneous
3	70	10	60	93.7 ± 2.7
4	60	20	60	98.0 ± 2.5
5	30	50	60	contaminated
6	-	100	60	8.4 ± 0.3
7	-	100	90	43.7 ± 2.7

[a]Conditions: OEGMA 20 vol%, 1 mM $CuBr_2$, $[MB^+]:[CuBr_2]:[TPMA]$ = 0.25:1:6, red light irradiation (λ_{max} = 625 nm, 4 mW cm^{-2}). [b]Measured by ellipsometry.

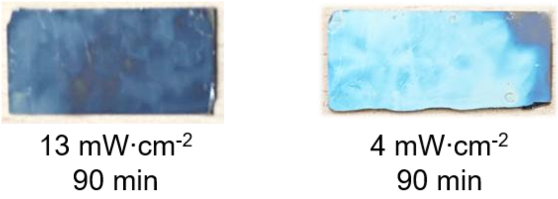

Figure S3. Photographs of the substrates after SI-photoATRP under red light in open air with varying light intensity. Polymerization conditions and brush thicknesses are reported in **Table 1** in the manuscript.

Table S3. Open-air SI-photoATRP of OEGMA under red light irradiation in the presence of EBiB as sacrificial initiator.[a]

**Entry**	**[EBiB] (mM)**	**time (min)**	**T_{dry} (nm)[b]**
1	5	60	8.7 ± 1.0
2	0.5	60	13.3 ± 1.0
3	0.25	90	20.8 ± 3.3
4	0.1	90	140 ± 12

[a]Conditions: OEGMA 20 vol%, DMSO 10 vol%, H_2O 70 vol%; 1 mM $CuBr_2$, $[MB^+]:[CuBr_2]:[TPMA]$ = 0.25:1:6, red light irradiation (λ_{max} = 625 nm, 4 mW cm^{-2}). [b]Measured by VASE.

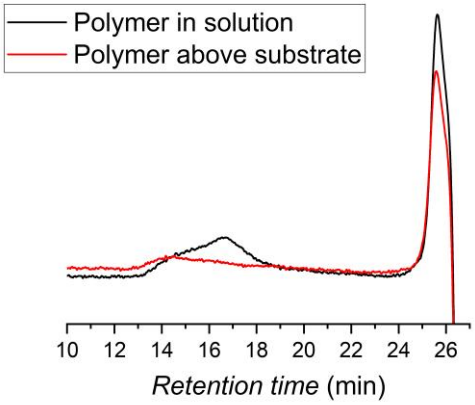

Figure S4. GPC traces of the polymerization mixture after 90 min irradiation, by withdrawing a small aliquot from the layer on top of the substrate (red line) and far from the substrate (black line). Polymerization conditions as in **Table S3**, Entry 4.

Table S4. Relevant parameters for SI-photoATRP systems on 2 x 1 SiO_x substrates (area = 2 cm^2) with varying the size of the Petri dish and reaction volume.

Entry	Volume mixture (mL)	S/V (cm^{-1})	Petri dish diameter (cm)	Solution height (mm)	T_{dry} (nm)[b]
1	5	0.4	5.7	1.6	93.7 ± 2.7
2	3	0.7	2.9	3.0	22.0 ± 1.7
3	1.5	1.3	2.9	0.8	110.3 ± 1.9
4	1.25	1.6	2.9	0.4	107.9 ± 2.0[c]

[a]Conditions: OEGMA 20 vol%, DMSO 10 vol%, H_2O 70 vol%; 1 mM $CuBr_2$, [MB^+]:[$CuBr_2$]:[TPMA] = 0.25:1:6; irradiated for 60 min under red light (λ_{max} = 625 nm, 4 mW cm^{-2}). [b]Measured by VASE. [c]After 90 min.

Table S5. Open-air SI-photoATRP of OEGMA under red light irradiation.[a]

Entry	[NaCl] (mM)	T_{dry} (nm)[b]
1	~140[c]	9.0 ± 4.0
2	140	1.4 ± 0.1
3	5	40.4 ± 3.8

[a]Conditions: OEGMA 20 vol%, DMSO 10 vol%, H_2O 70 vol%; 1 mM $CuBr_2$, [MB^+]:[$CuBr_2$]:[TPMA] = 0.25:1:6; irradiated for 90 min under red light (λ_{max} = 625 nm, 4 mW cm^{-2}). [b]Measured by VASE. [c]In PBS buffer (with 10 vol% DMSO).

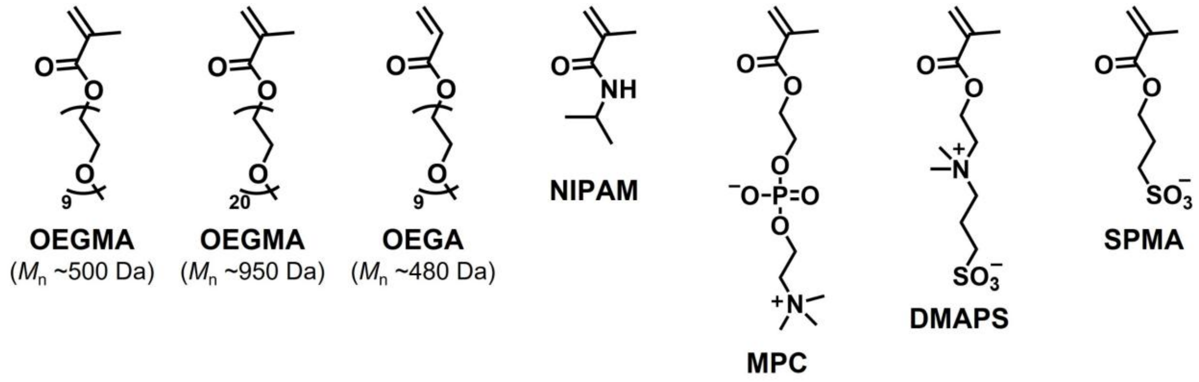

Figure S5. Chemical structures of the (macro)monomers employed to investigate the monomer scope of the open-air red light-mediated SI-photoATRP.

Table S6. Open-air SI-photoATRP of different monomers and light wavelengths.[a]

Entry	Light	λ_{max} (nm)	DMSO (vol%)	T_{dry} (nm)[b]
1	UV	365	10	74.4 ± 2.3[c]
2	UV	365	20	94.1 ± 3.2
3	Blue	420	10	2.4 ± 2.1
4	Green/Yellow	565	10	98 ± 11
5	NIR	780	10	1.2 ± 0.2

[a]Conditions: OEGMA 20 vol%, H_2O 70 vol%; 1 mM $CuBr_2$, [MB^+]:[$CuBr_2$]:[TPMA]:[NaBr] = 0.25:1:6:2; irradiated for 90 min under light at varying λ_{max}, but fixed intensity of 4 mW cm^{-2}. [b]Measured by ellipsometry. [c]Poor film homogeneity.

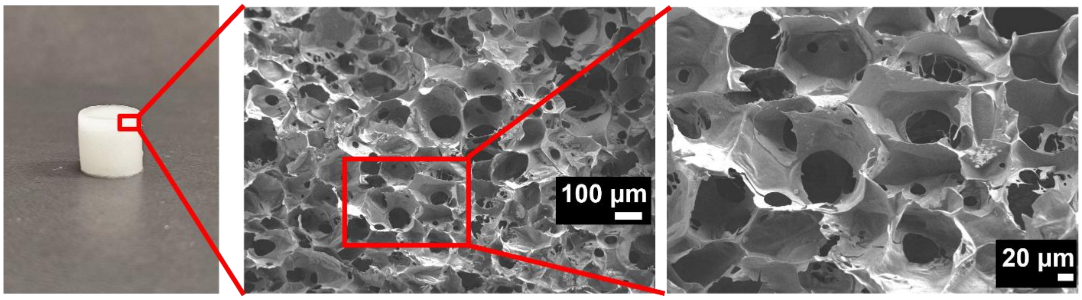

Figure S6. Photograph (left) and SEM pictures (middle, right) of the chitosan cryogel.

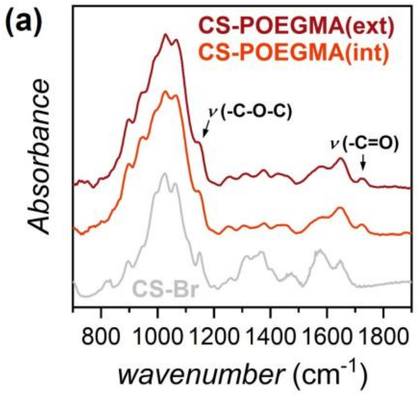

Figure S7. ATR-IR spectra showing the growth of POEGMA brushes inside the pores (interior, "int") and on the outer surface (exterior, "ext") of functional CS cryogels, by employing (a) red light, and (b) UV light. Conditions: OEGMA 20 vol%, H_2O 60 (UV)-70 (red) vol%; DMSO 10-20 vol%, 1 mM $CuBr_2$, $[MB^+]$:$[CuBr_2]$:[TPMA]:[NaBr] = 0.25:1:6:2; irradiated for 90 min at a fixed intensity of 4 mW cm^{-2}.

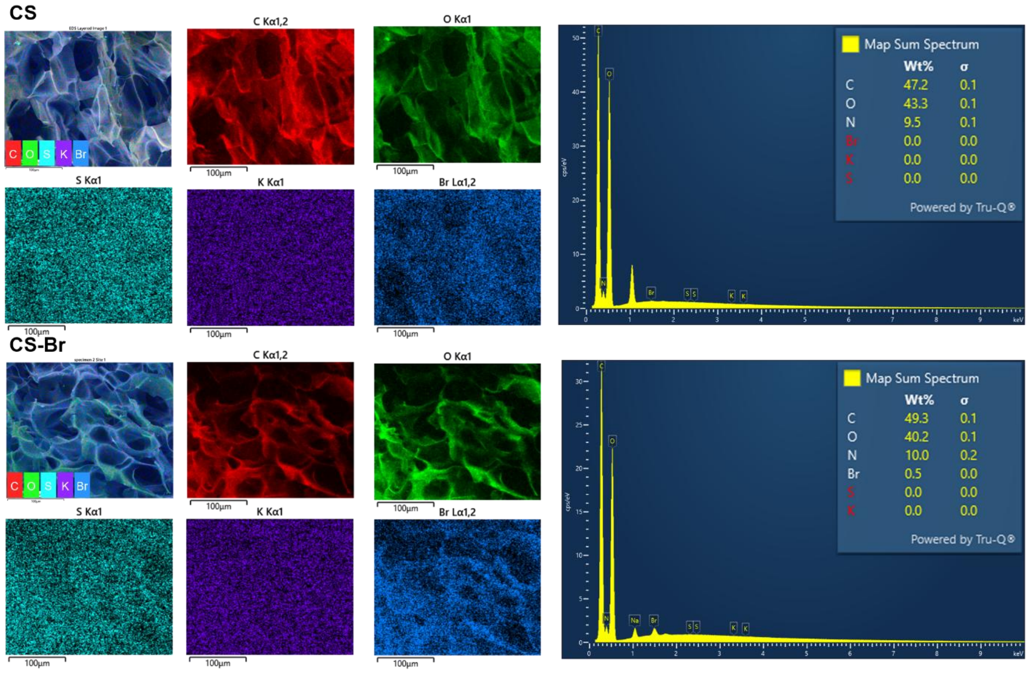

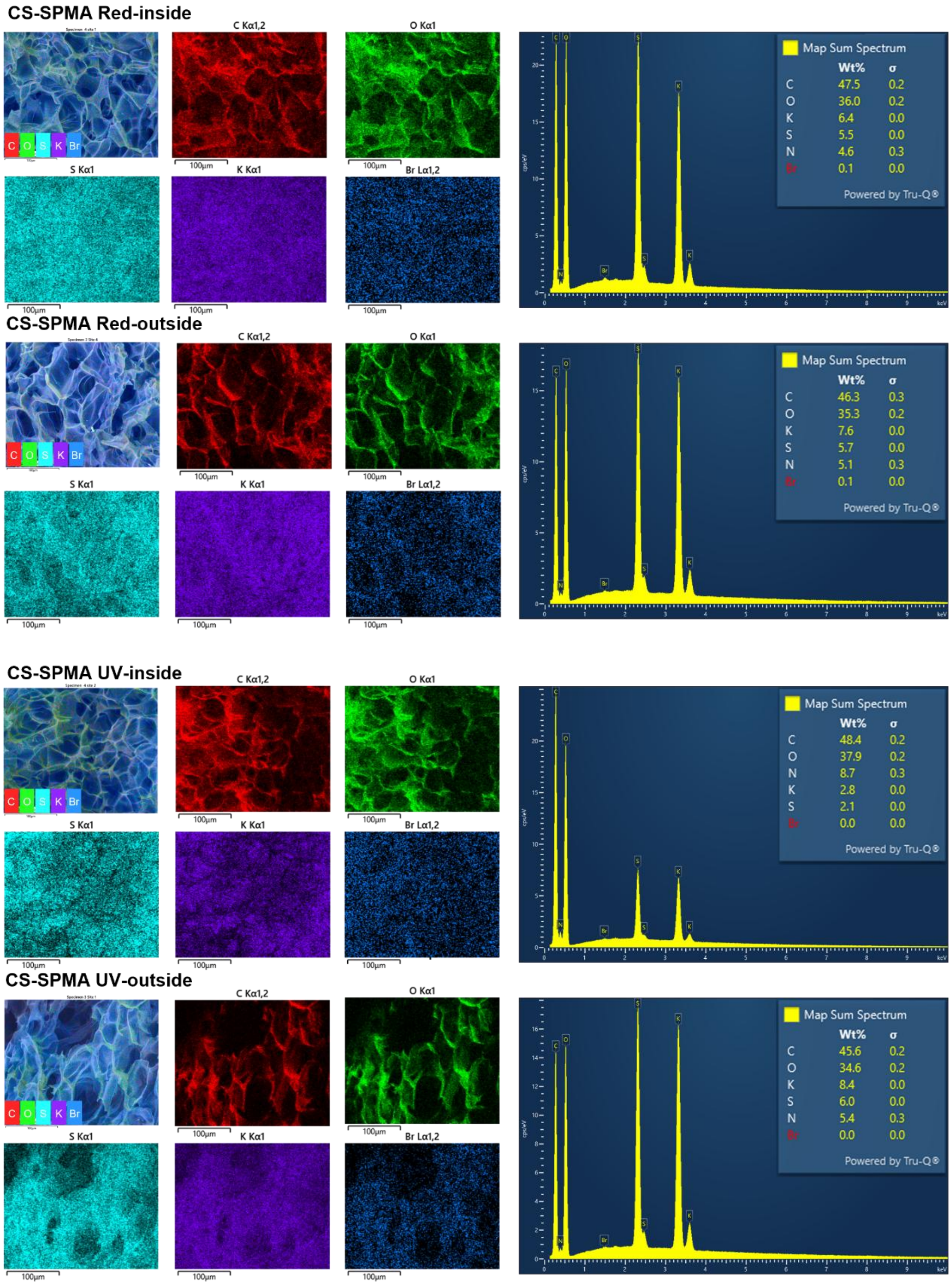

Figure S8. SEM-EDS mapping of CS cryogels highlighting the relative weight fraction of relevant elements: C, O, N, K, S. Potassium and sulfur are only present after the growth of PSPMA brushes. Different portions of the gels were cut and analyzed to evaluate the differences in brush growth on the interior of cryogels' porosities (labeled as "inside") and on the external surface (labeled as "outside"). From top to bottom: CS cryogel, CS cryogel functionalized with ATRP initiating sites, CS cryogels after the growth PSPMA brushes under red light irradiation (interior and external surface), CS cryogels after the growth PSPMA brushes under UV light irradiation (interior and external surface).

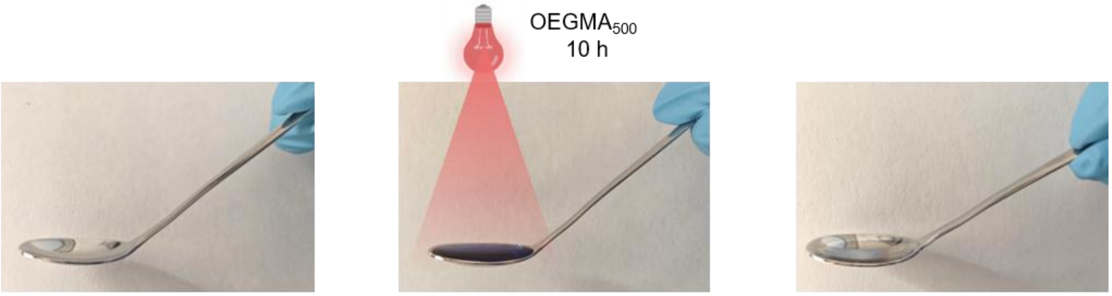

Figure S9. Photographs of the ATRP-initiator-functionalized spoon (left) filled with the SI-photoATRP polymerization mixture (middle) and rinsed after polymerization (right). Conditions: OEGMA 20 vol%, DMSO 10 vol%, H_2O 70 vol%; 1 mM $CuBr_2$, $[MB^+]$:$[CuBr_2]$:[TPMA] = 0.25:1:6; irradiated for 10 h under red light (λ_{max} = 625 nm, 4 mW cm^{-2}).
